# Early Alzheimer’s risk detection via diffusion tensor imaging using a few-shot multichannel attention residual learning network

**DOI:** 10.3389/frai.2026.1867451

**Published:** 2026-06-15

**Authors:** Arpit Shet, Saad Sabahuddin, Priyadarshini B, Bhanu Naga Sai Siva Ram Jami, John Sahaya Rani Alex, Chih-Yang Lin, Chi-Wen Lung

**Affiliations:** 1 School of Electronics Engineering, Vellore Institute of Technology, Chennai, India; 2 Department of Mechanical Engineering, National Central University, Taoyuan, Taiwan; 3 Department of Creative Product Design, Asia University, Taichung, Taiwan

**Keywords:** Alzheimer’s disease, attention networks, deep learning, diffusion tensor imaging, few-shot learning, mild cognitive impairment, residual learning

## Abstract

**Introduction:**

Alzheimer’s disease (AD) is responsible for many dementia-related deaths worldwide, and Mild Cognitive Impairment (MCI) is often its earliest clinical manifestation. Early detection of MCI is essential to initiate timely interventions that can slow progression and improve the patient’s quality of life. The emergence of diffusion tensor imaging (DTI) has made it a powerful neuroimaging modality for MCI detection, offering greater sensitivity to microstructural changes in white matter than basic structural imaging.

**Methods:**

A Multichannel Attention Residual Learning–DTI (MARL–DTI) architecture was designed with attention mechanisms and residual learning utilizing multiple DTI-derived diffusion metrics, including fractional anisotropy (FA), mean diffusivity (MD), radial diffusivity (RD), and axial diffusivity (AxD). To overcome the scarcity of labelled neuroimaging data, the model was supplemented with a few-shot learning framework. The dataset comprised 239 DTI scans from ADNI was used to evaluate the model. Local Interpretable Model-Agnostic Explanations (LIME) assists in analysing the model’s decision-making process behaviour.

**Results:**

The proposed MARL-DTI achieved a classification accuracy of 92.76%, outperforming baseline DenseNet variants, StatFusion-FCNN, and Matching Networks. Validation on an unseen dataset of 36 ADNI subjects yielded consistent accuracy (91.67%), confirming the model’s reliability. LIME analysis was used to provide instance-level explanations, enabling qualitative assessment of the model’s decision-making behaviour by highlighting feature contributions for individual predictions.

**Discussion:**

These results demonstrate that the proposed MARL-DTI framework surpasses traditional deep learning architectures in the early identification of MCI using DTI data. The model consistently achieved strong performance while maintaining transparency, using LIME as a validation tool to examine its decision-making behaviour. These findings highlight the potential of combining multi-channel volumetric DTI data with explainable deep learning approaches to support reliable and clinically relevant early detection of Alzheimer’s disease.

## 1 Introduction

Alzheimer’s disease (AD) is a progressive degenerative disease that results in the gradual loss of nerve cells, associated with memory and cognitive abilities (Silva et al., 2019). Based on World Health Organisation (WHO) report, around the globe, 55 million individuals are presently affected by dementia, with AD being a significant contributor (Dementia [Internet], 2025). As global life expectancy rises, the prevalence of AD is expected to increase substantially, highlighting the critical need for early detection to enable timely intervention and slow disease progression. Early diagnosis is the only way to improve patient outcomes and reduce the overall burden on healthcare systems.

Detection of AD at an early stage requires sensitive neuroimaging techniques capable of identifying subtle brain changes before clinical symptoms manifest. Diffusion Tensor Imaging (DTI) is an advanced imaging technique that captures microstructural alterations in white matter by quantifying water diffusion properties using biomarkers like Fractional Anisotropy (FA), Mean Diffusivity (MD), Axial Diffusivity (AxD), and Radial Diffusivity (RD) (Mielke et al., 2009; Oishi et al., 2011). These markers offer valuable insights of tissues health and can reveal early neurodegenerative changes, before visible brain atrophy occurs. Hence, as a non-invasive and widely accessible technique, DTI can provide subtle indicators of neurodegeneration long before other conventional imaging techniques reveal significant abnormalities (Mielke et al., 2009). Even though other neuroimaging methods are also commonly used in AD research, they have certain limitations in early detection. Like Structural MRI typically detects later-stage atrophy, whereas PET imaging, despite its effectiveness in detecting amyloid and tau pathology, is expensive, requires radioactive tracers, and has limited availability. Similarly, Functional MRI (FMRI), is used to assess brain function, but lacks in sensitivity needed to detect early structural degeneration. These limitations make DTI a more practical and sensitive choice for detecting the earliest pathological changes associated with AD progression (Oishi et al., 2011).

Hence advantage of DTI in detecting early microstructural changes associated with AD, allows the researchers to integrating this technique with advanced computational methods to enhance diagnostic accuracy and clinical usability. In particular, deep learning models have shown their effectiveness in automating AD diagnosis (Mohammed et al., 2024; Liu et al., 2022; Hechkel and Helali, 2025), with recent shifts toward lightweight architectures and few-shot learning approaches (Bazine et al., 2025) aimed at overcoming challenges posed by limited labelled neuroimaging data. Furthermore, recent advancements in deep learning for neuroimaging emphasise the importance of leveraging volumetric data representations to preserve spatial and anatomical information inherent in brain structures. Unlike models that rely on simplified feature representations fails to capture subtle microstructural variations associated with early neurodegeneration, so models that operates on full DTI volumes are needed. These developments highlight the increasing need for clinically relevant frameworks that can effectively learn from limited and heterogeneous medical datasets while maintaining high diagnostic performance (Khvostikov et al., 2018).

Many existing approaches focus only on FA and MD (Tang et al., 2016; Nir et al., 2014), ignoring the important insights from AxD and RD, which might also provide complementary information about axonal damage and demyelination. Further, despite their strength, deep Convolutional Neural Networks (CNN) and ensemble models are often computationally intensive, prone to overfitting, and less interpretable. These challenges limit their adoption in real-world clinical environments with limited computational resources and data availability.

The following are the main contributions of this study to address these limitations:

- Initial experiments employ MONAI-based DenseNet models on comprehensive volumetric DTI data to assess the effectiveness of deep convolutional networks when applied to high-dimensional neuroimaging inputs.
- Using statistical features from FA, MD, RD, and AxD, a statistical feature-based method (StatFusion-FCNN) is created that offers a computationally efficient solution for classification.
- Matching Networks are applied to the statistical feature space to enable few-shot learning, improving performance under limited labelled data conditions.
- A novel framework called Multichannel Attention Residual Learning (MARL-DTI) is proposed, which processes multiple volumetric DTI data (FA, MD, RD, and AxD) as a unified 4-channel 3D input. This preserves spatial and anatomical information while enabling the model to capture subtle white matter alterations, with spatial attention and residual learning enhancing feature representation and training stability.
- Prototypical Networks, which learn class prototypes in an embedding space and improve generalization from few samples, are included into the MARL-DTI system to overcome data scarcity and enable few-shot classification.

The proposed model MARL-DTI was evaluated with an unseen 18 MCI and 18 NC subjects from ADNI, that was entirely excluded from the training and validation phases. To verify the model’s behaviour, an instance-level explanation was generated using Local Interpretable Model-Agnostic Explanations (LIME) to validate the transparency of the model’s predictions. In summary, the present work proposes an integrated framework of volumetric DTI analysis, few-shot learning and attention-based modelling to facilitate early detection of MCI, while addressing the major challenges associated with data scarcity, model complexity and clinical applicability.

## 2 Materials and methods

### 2.1 Dataset description

This study utilises DTI data from the Alzheimer’s disease Neuroimaging Initiative (ADNI) (adni.loni.usc.edu), comprising 101 MCI subjects and 138 Normal Control (NC) subjects for training, testing, and validation. Subjects were included based on the availability of raw baseline DTI scans, and the age distribution criteria to be similar across both diagnostic groups. Within the MCI group, ages ranged from 55 to 99 years, with 35 females (34.7%) and 66 males (65.3%). The NC group ranged in age from 60 to 99 years, comprising 72 females (52.2%) and 66 males (47.8%). The overall dataset comprised 239 participants, including 107 females (44.8%) and 132 males (55.2%).

### 2.2 DTI preprocessing and metric extraction

A standardised preprocessing pipeline was used to minimise the noise, correct artefacts, and for accurate estimation of diffusion tensor metrics before feature extraction. The preprocessing begins with the conversion of Digital Imaging and Communications in Medicine (DICOM) files to Neuroimaging Informatics Technology Initiative (NIFTI) format, which is important for standardising medical imaging data. In contrast to DICOM files, which contain complex metadata and multi-slice formats, the NIFTI format simplifies storage, preserves spatial consistency, enables efficient preprocessing and analysis. Brain extraction was then performed on the non-diffusion-weighted (b0) image using the Brain Extraction Tool (BET) from the FMRIB Software Library (FSL v6.0) with a fractional intensity threshold of 0.2 to remove non-brain tissues like the skull and meninges (Smith, 2002). Eddy-current and motion correction were subsequently applied using the FSL eddy framework, where all diffusion-weighted image (DWI) volumes were aligned to the b0 reference image to minimise geometric distortions and inter-volume misalignment caused by eddy currents and subject motion (Andersson and Sotiropoulos, 2016). Following correction, diffusion tensor fitting was performed using DTIFIT in FSL to estimate voxel-wise diffusion tensors. The diffusion tensor is defined by three eigenvalues (*λ*₁, λ₂, λ₃), which correspond to the diffusion properties of water molecules along the principal directions. Based on these eigenvalues, the following scalar metrics were calculated:

Mean Diffusivity (MD): Reflects the overall rate of water diffusion and is calculated as shown in Equation 1.

$$\mathrm{MD}=\frac{\lambda_{1}+\lambda_{2}+\lambda_{3}}{3}$$ (1)

- Fractional Anisotropy (FA): Quantifies the directional coherence of water diffusion, reflecting the degree of white matter organisation and integrity, calculated as shown in Equation 2.

$$\mathrm{FA}=\sqrt{\frac{3}{2}}\cdot \frac{\sqrt{{\left(\lambda_{1}-\mathrm{MD}\right)}^{2}+{\left(\lambda_{2}-\mathrm{MD}\right)}^{2}+{\left(\lambda_{3}-\mathrm{MD}\right)}^{2}}}{\sqrt{\lambda_{1}^{2}+\lambda_{2}^{2}+\lambda_{3}^{2}}}$$ (2)

- Axial Diffusivity (AxD): Measures diffusion along the main axis of white matter fibres and is computed as shown in Equation 3.

$$\mathrm{AxD}=\lambda_{1}$$ (3)

- Radial Diffusivity (RD): Measures diffusion perpendicular to the fibres, indicating potential demyelination, and is defined as in Equation 4.

$$\mathrm{RD}=\frac{\lambda_{2}+\lambda_{3}}{2}$$ (4)

These metrics are essential for determining the white matter integrity and detecting early changes in MCI (Alexander et al., 2007). Finally, linear registration uses affine transformations to align all brain images to the Montreal Neurological Institute (MNI) 152 T1 2 mm template. This standardization guarantees consistent spatial alignment among participants, enabling reliable feature extraction for subsequent machine learning tasks. All preprocessing procedures were carried out using FSL, a widely used neuroimaging analysis toolkit.

### 2.3 DTI feature classification with deep neural networks

Four deep learning models were trained to classify subjects as MCI or NC using the extracted DTI features. The Primary objective was to gradually improve diagnostic accuracy while tackling challenges such as limited labelled data and inter-subject variability. Deep learning was chosen for its ability to automatically extract complex, non-linear patterns from high-dimensional DTI data, making it effective at detecting subtle brain changes associated with early cognitive decline. As a baseline, MONAI’s DenseNet (MONAI, 2022) a 3D adaptation of the conventional DenseNet architecture, specifically designed for medical imaging tasks was used. The model was employed to classify MCI and NC using four DTI-derived metrics (FA, MD, RD, and AxD).

#### 2.3.1 Statistical infused fully connected network (StatFusion-FCNN)

**Figure 1:**
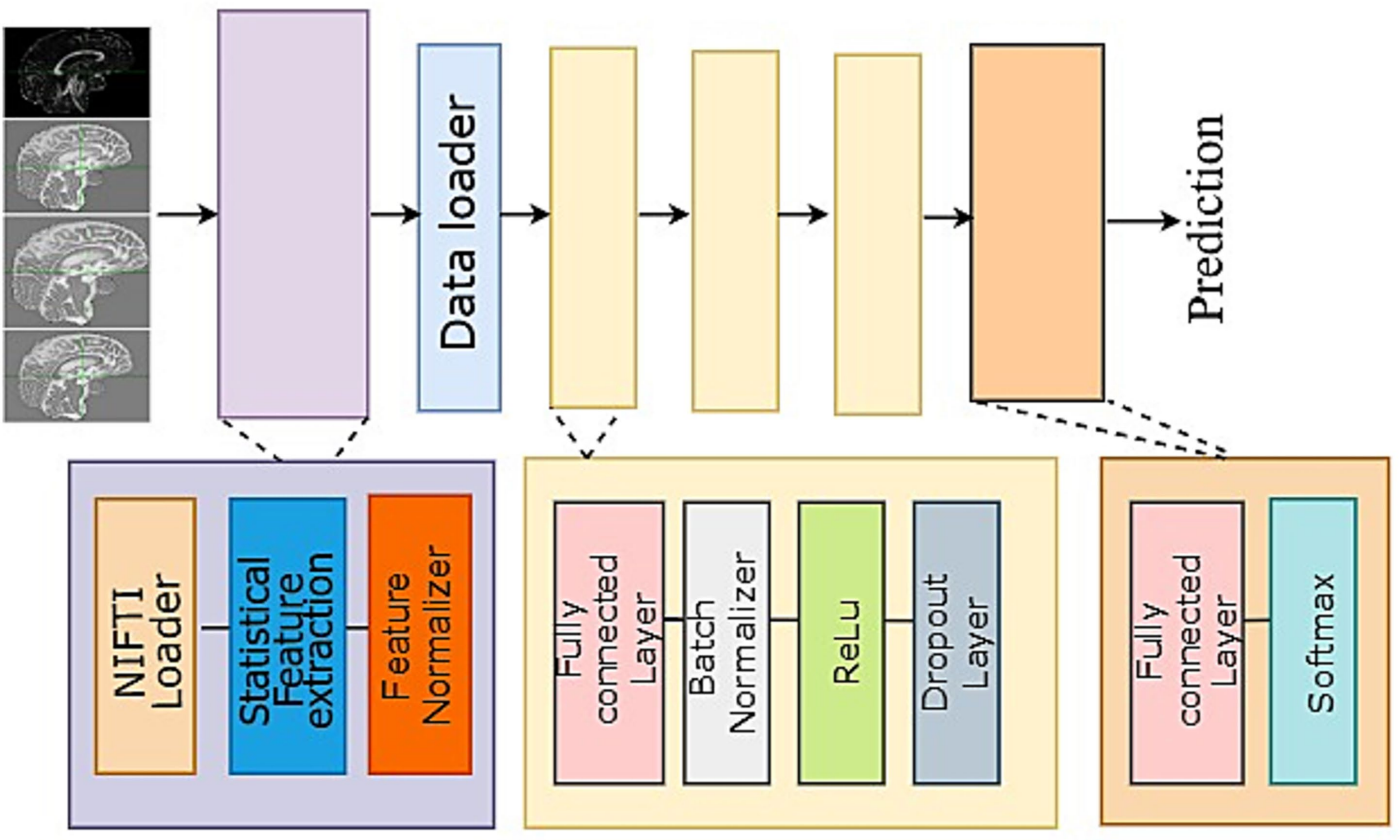
StatFusion-FCNN architecture.

Statistical Infused Fully Connected Network (StatFusion-FCNN) is a neural network designed for structured, low-dimensional inputs. Unlike CNNs, which capture spatial hierarchies in high-dimensional images, FCNNs operate on fixed-length feature vectors and learn global, non-linear patterns across all input dimensions (Zhou et al., 2020). Since the inputs are statistical descriptors extracted from brain-wide DTI maps (FA, MD, AxD, and RD), there is no spatial relationship to preserve. Thus, a simple and efficient FCNN is preferred over a CNN. The overall architecture of the StatFusion-FCNN model is depicted in Figure 1.

Statistical measures like the mean, standard deviation, percentiles, and extrema were derived for each individual’s DTI maps (FA, MD, AxD, and RD). Then, these statistical measures were concatenated into a 32-dimensional feature vector that represents the important global diffusion properties. To maintain consistency across subjects, the features were scaled using z-score normalisation (zero mean, unit variance). The normalised vectors were then fed into a series of fully connected layers. In order to improve the stability and mitigate overfitting, the fully connected layer is incorporated with Batch Normalisation, ReLU activation, and Dropout. Finally, a Softmax activation function was applied to produce class probabilities for the binary classification task of differentiating NC from MCI subjects.

#### 2.3.2 Matching networks

**Figure 2:**
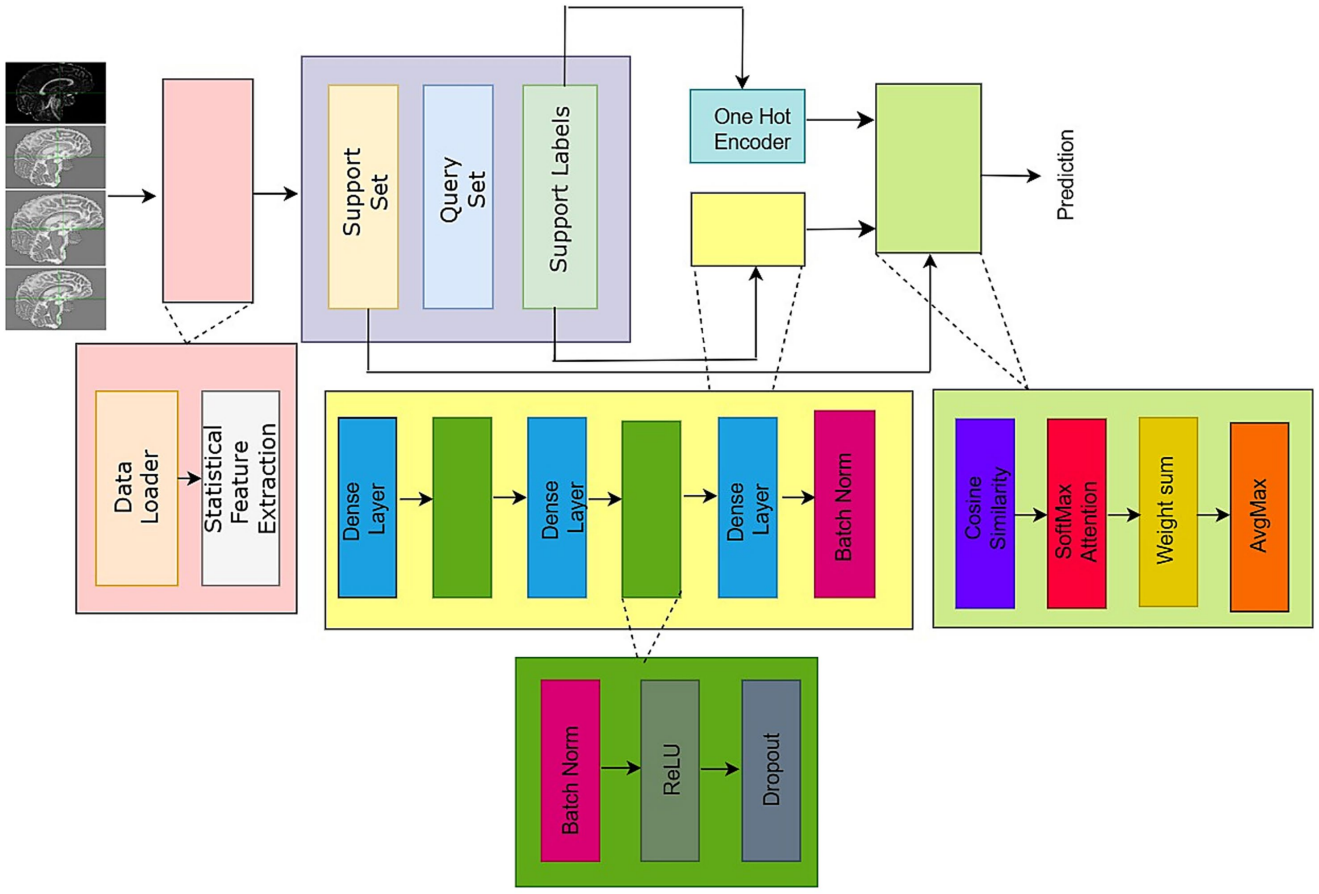
Architecture of the Matching Networks.

Initially, the traditional fully connected neural networks (FCNNs) were evaluated using statistical measures derived from DTI maps (FA, MD, AxD, and RD), as well as 3D CNN models were implemented with the MONAI framework. FCNNs effectively processed the 32-dimensional feature vectors but showed limited generalisation in low-data scenarios. In contrast, MONAI-based 3D CNNs captured spatial brain patterns but were computationally intensive and highly sensitive to preprocessing variations. Both models had difficulty in few-shot settings, where only a limited number of labelled samples were available. To overcome these challenges, we adopted Matching Networks (Vinyals et al., 2016), a metric-based non-parametric few-shot learning approach. The Matching Networks algorithm learns an embedding space where similar instances are clustered together, allowing for accurate classification even with a limited number of examples. The model compares the query samples to a small support set using attention-weighted similarity measures, making it well-suited for fine-grained distinctions in sparse clinical datasets. Its lightweight design and use of episodic training further enhance generalisation, mainly when operating on compact statistical representations. Figure 2 shows the architecture of the Matching Networks used for few-shot learning on DTI-metric-derived statistical features.

Each episode consists of a support set $S={\left\{(x_{i},y_{i})\right\}}_{i=1}^{N}$ containing N labelled samples and a query set $Q={\left\{x_{j}\right\}}_{j=1}^{M}$ with M unlabeled samples. For each DTI modality (FA, MD, AxD, RD), eight statistical features are extracted, including mean, median, minimum, standard deviation, maximum, and the 25th, 75th, and 90th percentiles. These features are concatenated across all modalities to form a 32-dimensional feature vector as expressed in Equation 5.

$$x=\text{concat}([FA_{\text{stats}},MD_{\text{stats}},\mathrm{Ax}D_{\text{stats}},RD_{\text{stats}}])\in R^{32}$$ (5)

The resulting feature vector is then fed into an embedding network composed of fully connected layers with batch normalisation, ReLU activation, and dropout, which transforms the input into a compact d-dimensional representation. During classification, cosine similarities between each query embedding and the support set embeddings are computed. These similarity scores are converted into attention weights using a softmax function, which are later used to generate the predicted label as a weighted combination of the support labels. The final class assignment is based on the label with the highest probability in the predicted label distribution.

#### 2.3.3 Proposed prototypical networks with multi-channel attention residual learning (MARL DTI)

Prototypical Networks uses a distance-based classification approach that is especially effective in few-shot learning scenarios where labelled data are scarce (Snell et al., 2017). Unlike Matching Networks, which compare a query sample with every support sample, Prototypical Networks summarise each class using a single representative vector called a prototype. The prototype vector is obtained by averaging the features of all support samples belonging to that class. During classification, the query sample is assigned to the class whose prototype is closest to it in the feature space.

Determined by the limitations of our previous methods that statistical feature representations and baseline architectures have limited ability to capture subtle white matter abnormalities in volumetric DTI data. Hence, a novel Multi-channel Attention Residual Learning DTI (MARL-DTI) framework was proposed to improve early MCI classification. Instead of employing low-dimensional statistical features, the proposed model directly utilises volumetric DTI data, maintaining spatial and anatomical information essential for detecting subtle white matter changes.

In this framework, each subject is represented by four preprocessed DTI maps (FA, MD, AxD, and RD), which are combined into a 4-channel 3D input. That allows the model to capture complementary diffusion information from different DTI maps. A 3D CNN is used as the feature encoder to extract meaningful representations from these inputs.

The feature encoder is comprised of an initial Conv3D layer (32 filters, kernel size of 3), followed by Batch Normalisation and Leaky ReLU activation for low-level feature extraction. This is followed by three residual blocks where the channel dimensions are gradually increased from 32 to 64, from 64 to 128, and from 128 to 256, to learn hierarchical features. After each residual block, max-pooling is applied to reduce spatial dimensions and to improve the computational efficiency. A spatial attention module is introduced between the second and third residual blocks to improve feature discrimination. This module comprises two sequential 1 × 1 Conv 3D layers: the first reduces the number of channels, and the second generates a single-channel attention map, followed by a Sigmoid activation. The attention map is applied via element-wise multiplication to emphasise clinically relevant regions. Finally, global average pooling and global max pooling are applied, and their outputs are combined to form a compact feature vector. The feature vector is regularised through dropout, with a rate of 0.3 before being fed into a fully connected layer, which results in a 256-dimensional embedding. This embedding then serves as the basis for classification using prototypes.

##### 2.3.3.1 Episodic training framework

The model is trained in an episodic few-shot learning approach. Each episode includes two sets: a support set with labelled examples from each class and a query set to assess the model’s predictions. A shared encoder network converts all input volumes into a latent embedding space. For each class, a prototype is calculated as the average of the embeddings of its support samples, shown in Equation 6.

$$c_{k}=\frac{1}{|S_{k}|}\sum_{x_{i}\in S_{k}}f(x_{i})$$ (6)

where $S_{k}$ represents the set of support samples belonging to class k , and $f(x_{i})$ denotes the embedding of input $x_{i}$ . During classification, each query sample is mapped to the same embedding space and compared with the class prototypes. The prediction is made based on the minimum squared Euclidean distance, as shown in Equation 7.

$$d(x,c_{k})=∥f(x)-c_{k}{∥}^{2}$$ (7)

**Figure 3:**
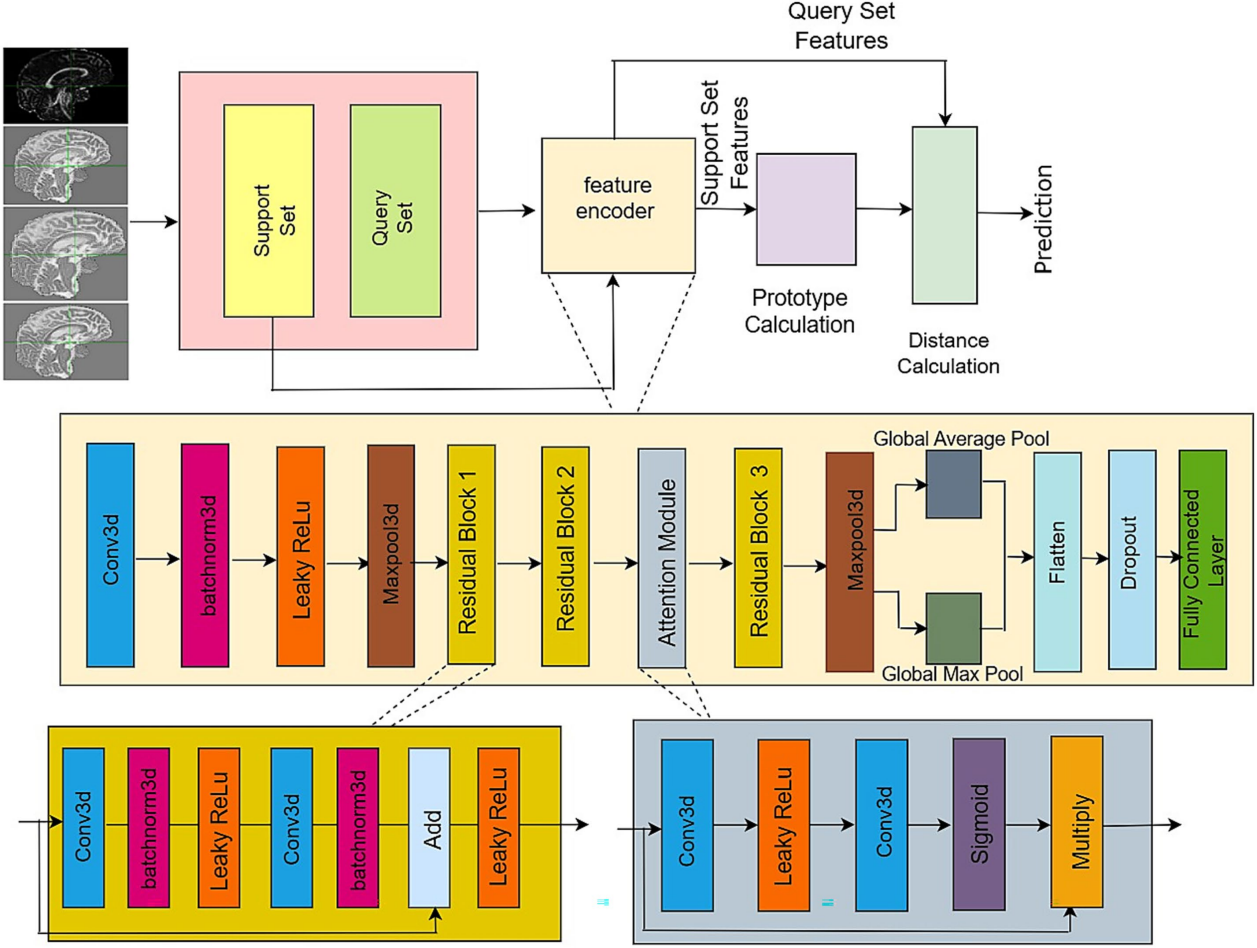
Proposed MARL DTI architecture.

The integration of spatial attention mechanisms allows the model to focus on significantly relevant regions, while residual connections support stable training of deeper architectures by enhancing gradient flow. The complete architecture of the proposed MARL-DTI model is depicted in Figure 3.

## 3 Implementation details

All models are implemented using PyTorch 2.2.2 and trained on RTX A4000 GPU. The models were optimized using either the Adam or AdamW optimiser, with an initial learning rate of 0.001. Cross-Entropy Loss was used for all classification tasks. Training was performed for 100 epochs with early stopping based on validation performance to reduce overfitting. A dropout rate of 0.3–0.4 was applied for regularization, and batch sizes ranged from 4 to 16 depending on model architecture and GPU memory constraints.

The primary dataset consisted of 239 subjects for model development, including episodic training and internal validation. The support and query sets were dynamically sampled during episodic learning with subject-level separation to prevent data leakage to evaluate the robustness and generalization of the model, a separate, independent cohort of 36 completely unseen subjects was used, which was stored separately from the main dataset and exclusively for the final testing and evaluation. Fixed random seeds (seed = 42) were applied across Python, NumPy, and PyTorch implementations to ensure reproducibility of dataset partitioning and model evaluation.

MARL-DTI is based on a 2-way K-shot episodic learning setting in the few-shot learning setting. During each training episode, the support set consisted of *K* = 5 randomly sampled subjects per class (MCI and NC), while the query set contained 15 subjects per class selected from the remaining training pool without replacement. 100 episodes were generated per epoch for 20 training epochs. During evaluation, 10 support samples were randomly selected for each class from the hold-out support pool, and class prototypes were obtained by averaging support embeddings of each class, to classify query samples based on Euclidean distance to class prototypes. To improve robustness and reduce sampling bias, the evaluation procedure was repeated across five independent runs using different randomly sampled support sets. Final performance metrics, including accuracy, precision, recall, and F1-score, were reported as mean ± standard deviation across runs. The primary training dataset was used to select hyperparameters, like learning rate, dropout rate and embedding dimension and a separate cohort of 36 subjects was held out for final evaluation.

## 4 Results

### 4.1 Preprocessing results

**Figure 4:**
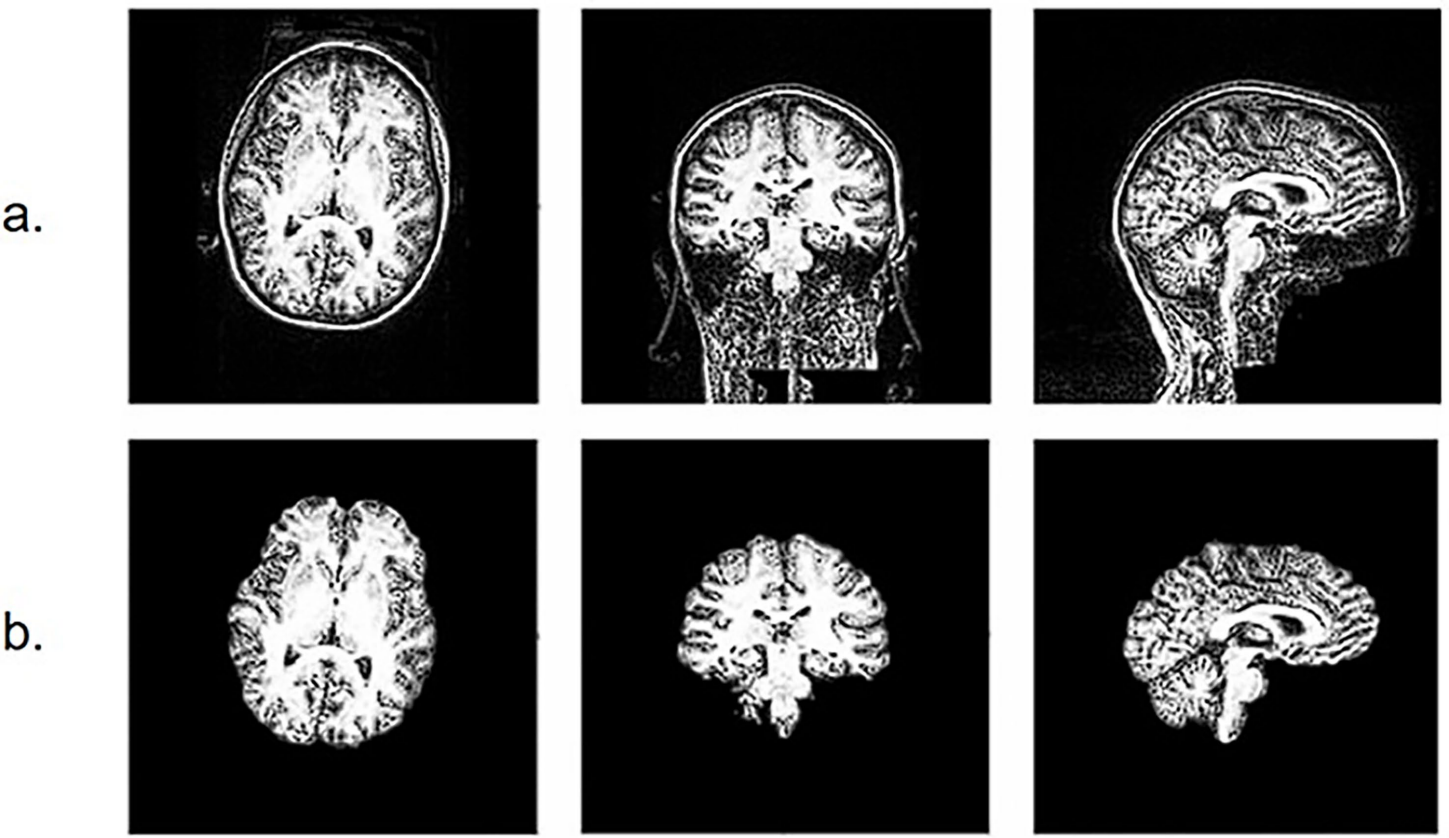
Brain extraction **(a)** Original image. **(b)** Skull-stripped output image showing only brain tissue.

**Figure 5:**
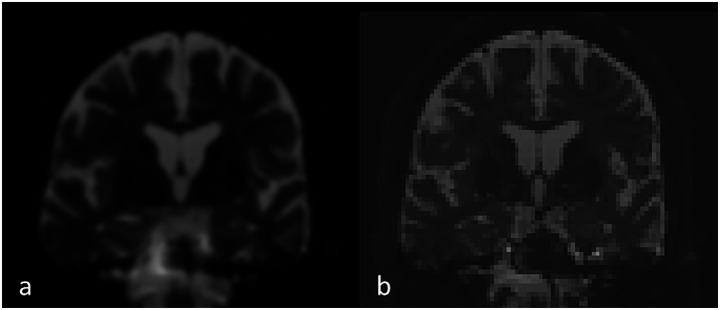
Motion and Eddy Current Correction Outcome. **(a)** Uncorrected DTI scan **(b)** Corrected scan with improved clarity.

**Figure 6:**
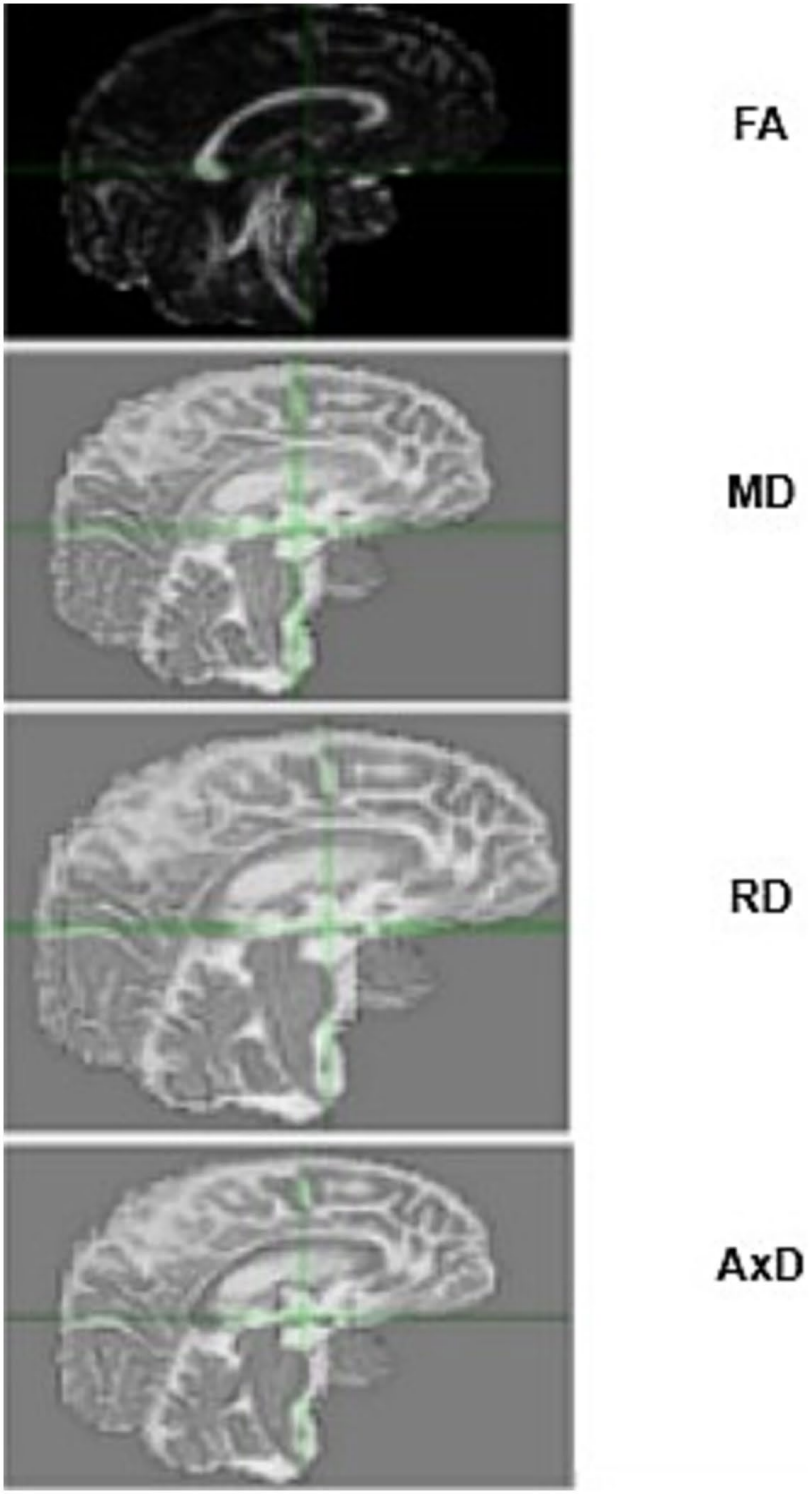
Diffusion metric maps.

Preprocessing is necessary for the reliable and accurate analysis of diffusion MRI. Raw diffusion images includes non-brain tissue, motion related distortions, and spatial inconsistencies that can negatively impact model performance and anatomical interpretation. To eliminate these issues, a standardised preprocessing pipeline was implemented, which includes skull stripping, motion and eddy current correction, tensor fitting, and spatial normalisation. These preprocessing pipeline ensures that only relevant brain structures are retained, reduces image distortions, and aligns all scans to a common reference space, thus allowing for consistent analysis across subjects. In Figure 4 the original diffusion image in row (a), which contains non-brain tissues such as the skull and meninges, whereas the image in row (b) clearly demonstrates their successful removal, retaining only the brain parenchyma is shown. This confirms the effective isolation of relevant anatomical structures for downstream diffusion analysis. Further, Figure 5 shows the output of motion and eddy-current correction, where image (a) represents the distorted raw DWI prior to correction and image (b) shows the corrected image aligned to the corresponding b0 reference image with improved structural consistency and reduced geometric distortion. Finally the derived FA, MD, AxD, and RD maps are shown in Figure 6 confirming the successful voxel-wise estimation of diffusion tensor properties from the preprocessed DTI data.

### 4.2 Performance of deep learning models

This section presents the classification performance of multiple deep learning models designed to classify MCI and NC. Four models were evaluated: baseline MONAI-based DenseNet variants, the StatFusion-FCNN, Matching Networks, and the proposed MARL-DTI framework. Performance of the deeplearning models was measured using accuracy, precision, recall, and F1-score.

#### 4.2.1 MONAI DenseNet

To evaluate the performance of deep convolutional networks for a classification task, multiple DenseNet variants were implemented from MONAI. As shown in Table 1, the highest accuracy of 61.22% was achieved by DenseNet201, followed by DenseNet169 (60.25%). These results suggest that moderately deep architectures provide a better trade-off between learning capacity and generalisation on limited datasets. On the other hand, DenseNet264 reached a lower accuracy (55.23%) possibly due to overfitting or optimization issues related to increased depth. Overall, these results emphasize the importance of balancing model complexity in the context of small neuroimaging datasets.

**Table 1:** Comparison of MONAI DenseNet models with different hyper parameters.

Model	Batch size	Learning rate	Weight decay	Accuracy (%)
DenseNet121	4	0.001	0.00	58.33
DenseNet169	8	0.002	0.20	60.25
DenseNet201	4	0.002	0.10	61.22
DenseNet264	4	0.001	0.01	55.23

#### 4.2.2 StatFusion-FCNN

The StatFusion-FCNN achieved significantly better classification performance compared to the DenseNet variants. It achieved an overall accuracy of 81.59%, which is more than the best DenseNet model (DenseNet201). The FCNN was particularly effective at identifying NC subjects, with a precision of 0.81, a recall of 0.90, and an F1 Score of 0.85. The model achieved a recall of 0.70, a precision of 0.84, and an F1 score of 0.70 but the performance was slightly worse on MCI. It still outperformed the DenseNet models, which showed greater limitations in distinguishing early cognitive decline. These results highlight the FCNN’s ability to learn effectively from low-dimensional, statistically derived DTI features. The performance metrics of StatFusion FCNN are shown in Table 2.

**Table 2:** Performance metrics of StatFusion FCNN.

Class	Precision	Recall	F1-score	Accuracy
NC	0.81	0.90	0.85	81.59%
MCI	0.84	0.70	0.76	

#### 4.2.3 Matching networks

Matching Networks performed better than the other two models, StatFusion-FCNN and the DenseNet models, in terms of classification performance, as shown in Table 3. Achieving an overall accuracy of 87.87%, Matching Networks outperformed the FCNN (81.59%) and significantly exceeded the best-performing DenseNet variant (61.22%). In terms of class-wise analysis, Matching Networks exhibited considerably higher precision of 0.90 and F1-score of 0.85 for the MCI class compared to FCNN. Moreover, it outperformed all DenseNet configurations across every evaluated metric. In spite of its better performance, the reduced recall of Matching Networks for MCI cases highlighted its difficulty in detecting early cognitive decline using statistical features derived from DTI maps. Matching Networks achieved a higher recall (0.80 vs. 0.70). This indicates greater sensitivity and a lower false negative rate for subtle MCI patterns in DTI-derived features.

**Table 3:** Performance metrics of matching networks.

Class	Precision	Recall	F1-score	Accuracy
NC	0.87	0.93	0.90	87.87%
MCI	0.90	0.80	0.85	

#### 4.2.4 Multi-channel attention residual learning DTI (MARL-DTI)

The proposed MARL-DTI achieved the highest classification performance, exceeding all evaluated models, with an overall accuracy of 92.76%, surpassing DenseNet (61.22%), StatFusion-FCNN (81.59%), and Matching Networks (87.87%). Besides the higher accuracy, MARL-DTI also achieved high precision (97.42%) and F1-score (92.09%), which indicates its predictions are reliable and balanced for both classes. For MCI detection, the model achieved 88.00% recall, which is better than Matching Networks (80.00%) and StatFusion-FCNN (70.00%), showing better sensitivity to early cognitive decline. The superior performance of MARL-DTI is attributed to its capability of directly exploiting multi-channel volumetric DTI data, where spatial and anatomical information were preserved that is lost in statistical feature representations. Then by integrating FA, MD, AxD, and RD maps as a unified 3D input, the model captures subtle microstructural variations across white matter regions. In addition, the attention mechanisms and residual learning enables the model to focus on the most informative regions while maintaining stable and efficient training. This combination allows MARL-DTI to learn more discriminative and clinically relevant representations, resulting in improved classification performance and generalization. The Classification Performance of MARL-DT is shown in Table 4.

**Table 4:** Classification performance of MARL-DTI.

Class	Precision	Recall	F1-score	Accuracy
NC	0.8902	0.9733	0.9297	0.9276
MCI	0.9706	0.8800	0.9230	

##### 4.2.4.1 Validation of model predictions

**Figure 7:**
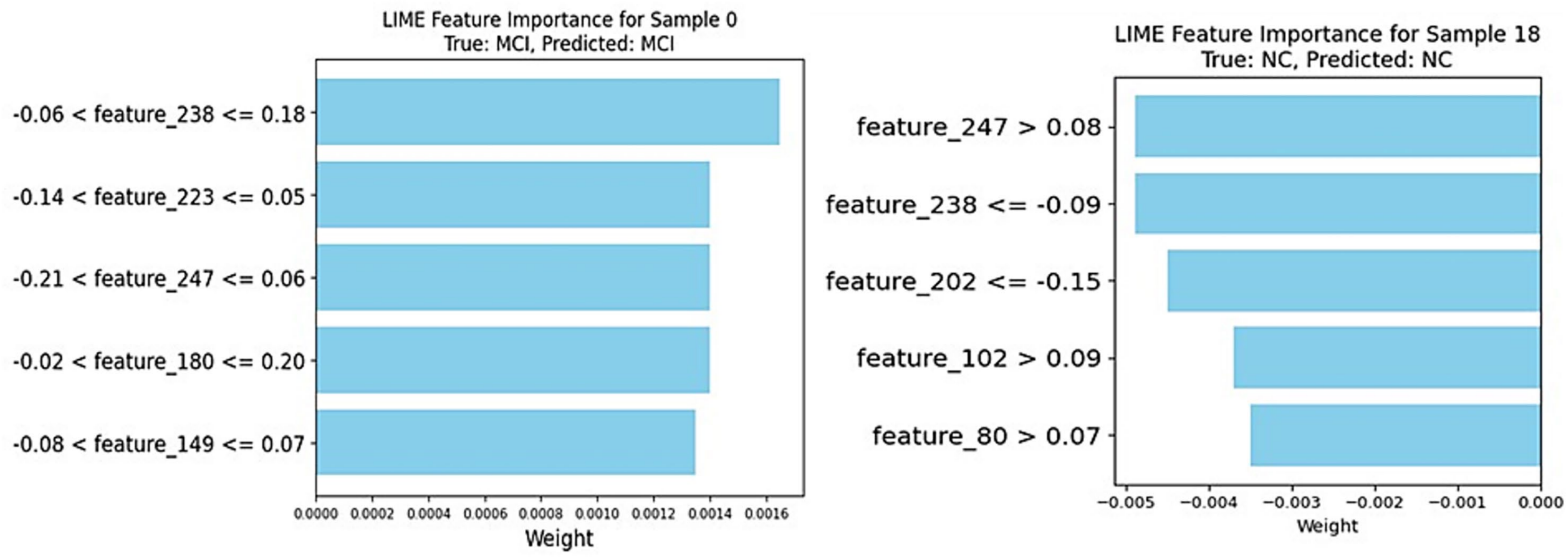
LIME-based feature importance for sample-level predictions in MARL-DTI.

In order to evaluate the relability of the model, the proposed MARL-DTI framework was further validated on an independent cohort of 36 completely unseen subjects, achieving an accuracy of 91.67%, consistent with the primary evaluation results. To improve transparency and to investigate whether or not the model was learning meaningful feature patterns or making random predictions (Ribeiro et al., 2016), Local Interpretable Model-Agnostic Explanations (LIME) was employed to examine individual model decisions. The generated explanations showed consistent patterns of feature contribution for correctly classified MCI and NC samples, indicating that the predictions were based on structured learned representations rather than arbitrary decisions. Figure 7 shows representative LIME visualizations with positive and negative feature weights showing how different input features affect classification results.

### 4.3 Comparative analysis of DTI-based deep learning model performances for MCI vs. NC classification

The recent studies that employs deep learning to classify MCI and NC using various neuroimages are summarised in Table 5. This comparative assessment highlights both unimodal and multimodal approaches, with a special emphasis on DTI. Our proposed model, built exclusively on DTI-derived features (FA, MD, RD, and AxD), achieves a high classification accuracy of 92.76% despite relying on a single imaging modality. This performance either exceeds or closely matches that of more complex multimodal frameworks.

**Table 5:** Performance of deep learning models for the classification of MCI vs. NC using neuroimaging modalities.

Study	Model type	Modalities used	Accuracy (MCI vs NC)
MARL-DTI	Custom deep model	DTI (FA, MD, RD, and AxD)	92.76%
Aderghal et al. (2020)	CNN + transfer learning	DTI (MD) + T2 MRI (hippocampus)	78.48%
Marzban et al. (2020)	CNN (shallow)	DTI (MD) + T1 MRI (GM maps)	79.6%
Kang et al. (2020)	CNN (comparison)	DTI (FA only)	67.5%
		DTI (MD only)	71.7%
Liu et al. (2018)	3D + 2D CNN (cascaded)	sMRI + FDG-PET	82.95%
Suk et al. (2014)	Deep boltzmann machine	sMRI + PET (voxel-level features)	85.67%

For instance, Aderghal et al. (2020) used cross-modal and cross-domain transfer learning on MD maps produced from DTI with sMRI and MNIST data, achieving 78.48% accuracy after fusion. Marzban et al. (2020) used DTI (MD) with T1-based grey matter maps with a shallow CNN and reported 79.6% accuracy. Kang et al. (2020), focused on early MCI (EMCI), reported 67.5 and 71.7% accuracy using FA and MD individually, and 94.2% when fusing both with sMRI, though this involved significant complexity. Liu et al. (2018) and Suk et al. (2014) used sMRI and PET in cascaded CNNs and Deep Boltzmann Machines, reaching 82.95 and 85.7%, respectively. Such comparisons demonstares the effectiveness of the proposed DTI-only framework which obtain strong predictive performance while reducing the need for complex multimodal data acquisition and preprocessing. The model also demonstrates reliable prediction behaviour and potential for practical clinical applications.

## 5 Discussion

The objective of the present study was to investigate the potential of deep learning models for early detection of MCI using DTI-derived biomarkers. After evaluating different architectures and applying standard preprocessing pipeline, key insights were gained. The quality and homogeneity of the imaging data were hugely improved by procedures such as skull stripping, motion correction and spatial normalisation. This improvement in consistency leds the model to be more stable and generalizable performance when trained on preprocessed data.

Although conventional deep learning models like DenseNet have been successful in natural image tasks, where found to struggle with generalisation in the medical imaging domain with limited data. These models were computationally expensive but also sensitive to variations in input format and data scarcity, which limits their practical applicability in clinical contexts. This finding strengthens a broader theme in medical AI research: increased depth and complexity do not always lead to better results, especially in low-data environments.

In terms of limited data, StatFusion-FCNN model that operates on structured feature representations achieved better performance than conventional CNNs, highlighting the importance of feature representation in low-data settings. Further, Matching Networks along with few-shot learning paradigm improved performance by capturing the similarities between samples and improve classification with limited training data. The proposed MARL-DTI framework extends these advantages by directly leveraging multi-channel volumetric DTI data within a few-shot learning setting. By integrating FA, MD, AxD, and RD maps into a unified 3D input, the model preserves spatial and anatomical information which was not captured by simplified feature representations. The intergartion of attention mechanisms and residual learning enables the model to focus on informative regions, thereby improving sensitivity to subtle microstructural changes associated with early cognitive decline. This results in more discriminative feature learning and superior classification performance.

## 6 Conclusion

Early detection of MCI is essential for timely intervention in AD, but identifying subtle microstructural brain changes remains challenging. To address this, this study developed a MARL-DTI,a deep learning framework that utilises multichannel diffusion tensor imaging (DTI) metrics, including fractional anisotropy, mean diffusivity, radial diffusivity, and axial diffusivity. The Process starts with pre processsing of DTI, then extracting multi-parametric diffusion features, and feeding them into a unified architecture incorporating attention mechanisms and residual learning to capture fine-grained white matter alterations. To address limited labelled data, a few-shot learning strategy was adopted, enabling efficient learning from small datasets. MARL-DTI was able to achieve 92.76% accuracy and 91.67% on an unseen test set, showing good generalisation. A comparative evaluation showed that MARL-DTI performeb better thasn baseline models, including DenseNet variants, StatFusion-FCNN, and Matching Networks, highlighting its superior feature representation. In addition, LIME-based was used to provide instance-level explanations, offering an insights on model’s prediction. This study offers a comprehensive and efficient pipeline that covers DTI preprocessing, feature extraction, and prediction validation. It shows that integrating multichannel DTI data with attention-based, data-efficient deep learning provides a reliable, clinically trustable approach for early detection of Alzheimer’s disease.

## Data Availability

The data used in this study were obtained from the Alzheimer’s Disease Neuroimaging Initiative (ADNI), which is publicly available at http://adni.loni.usc.edu.
